# Hormone-regulated expression and distribution of versican in mouse uterine tissues

**DOI:** 10.1186/1477-7827-7-60

**Published:** 2009-06-05

**Authors:** Renato M Salgado, Luciane P Capelo, Rodolfo R Favaro, Jocelyn D Glazier, John D Aplin, Telma MT Zorn

**Affiliations:** 1 Laboratory of Reproductive and Extracellular Matrix Biology, Department of Cell and Developmental Biology, Institute of Biomedical Sciences, University of São Paulo, São Paulo, Brazil; 2 Maternal and Fetal Health Research Group, School of Clinical and Laboratory Sciences, University of Manchester, Manchester, UK

## Abstract

**Background:**

Remodeling of the extracellular matrix is one of the most striking features observed in the uterus during the estrous cycle and after hormone replacement. Versican (VER) is a hyaluronan-binding proteoglycan that undergoes RNA alternative splicing, generating four distinct isoforms. This study analyzed the synthesis and distribution of VER in mouse uterine tissues during the estrous cycle, in ovariectomized (OVX) animals and after 17beta-estradiol (E2) and medroxyprogesterone (MPA) treatments, either alone or in combination.

**Methods:**

Uteri from mice in all phases of the estrous cycle, and animals subjected to ovariectomy and hormone replacement were collected for immunoperoxidase staining for versican, as well as PCR and quantitative Real Time PCR.

**Results:**

In diestrus and proestrus, VER was exclusively expressed in the endometrial stroma. In estrus and metaestrus, VER was present in both endometrial stroma and myometrium. In OVX mice, VER immunoreaction was abolished in all uterine tissues. VER expression was restored by E2, MPA and E2+MPA treatments. Real Time PCR analysis showed that VER expression increases considerably in the MPA-treated group. Analysis of mRNA identified isoforms V0, V1 and V3 in the mouse uterus.

**Conclusion:**

These results show that the expression of versican in uterine tissues is modulated by ovarian steroid hormones, in a tissue-specific manner. VER is induced in the myometrium exclusively by E2, whereas MPA induces VER deposition only in the endometrial stroma.

## Background

The estrous cycle is orchestrated by ovarian sex hormones [1]. In the mature mouse, estrogen (E2) produced during estrus stimulates epithelial cell proliferation and synthesis of progesterone receptors (PR). On the other hand, progesterone (P4) inhibits epithelial proliferation while stimulating the multiplication of stromal cells that characterizes the beginning of decidualization [2,3]. The combined action of E2 and P4 prepares uterine tissues for blastocyst implantation.

Estrogen receptors (ERα and ERβ) and progesterone receptors (PRA, PRB and PRC) are transcription factors that regulate gene expression by direct binding to DNA regulatory sequences and by specific interactions with co-activators and/or co-repressor proteins [4,5]. In response to the normal changes in the levels of E2 and P4, the endometrium and myometrium undergo extensive cellular and extracellular modification [6].

The ECM is a complex structure of macromolecules capable of self-assembly and is composed predominantly of collagens, non-collagenous multiadhesive glycoproteins, elastin, hyaluronan and proteoglycans [7]. The endometrial ECM plays important roles in endometrial decidualization, embryo implantation, trophoblast cell invasion and the maintenance of gestation [8-10]. Previous reports have documented the remodeling of collagen [11-14], as well as glycosaminoglycans and proteoglycans in the mouse uterus during early pregnancy [15,16]. Salgado *et al*. [17] have shown the differential distribution of four members of the small leucine-rich proteoglycan (SLRP) family in mouse endometrium and myometrium during the estrous cycle, suggesting that their expression in the uterine ECM is modulated by ovarian hormones. San Martin *et al*. [18] also detected hyaluronan (HA) and versican (VER) in endometrial stroma during the peri-implantation period, when angiogenesis, cell migration, trophoblast invasion and cell proliferation occur. After implantation, HA disappeared from the decidual region immediately surrounding the implantation chamber, whereas VER accumulated in the same region, suggesting this proteoglycan plays a role in proliferation and differentiation of endometrial fibroblasts into decidual cells, and may influence trophoblast invasion.

VER is a large chondroitin sulfate proteoglycan that belongs to the family of hyaluronan-binding proteoglycans termed hyalectins, and is found in many soft tissues. The middle region of the core protein is encoded by two large exons that specify the chondroitin sulfate attachment regions [19,20]. RNA splicing occurs in the two GAG attachment domains, encoded by exons 7 (αGAG) and 8 (βGAG), giving rise to four distinct isoforms termed V0, V1, V2 and V3. V0 possesses both exons 7 and 8, V1 only exon 8, V2 only exon 7 and V3 possesses neither [21]. The interaction between VER and hyaluronan (HA) is mediated by the N-terminal G1 globular domain, while the carboxy-terminal globular domain (G3) consists of a C-type lectin adjacent to two epidermal growth factor domains and a complement regulatory region. VER interacts with other ECM molecules, such as tenascin-R, collagen I, fibronectin and the elastic fiber-associated proteins fibrillin-1 and fibulin-2 [22]. Fibrillin-1 is present in the endometrial stroma in estrus and diestrus [23]. In addition, VER binds to chemokines and cell surface receptors including β_1_-integrin, CD44, epidermal growth factor receptor and selectins [24]. These interactions facilitate essential biological processes, including cell migration [20].

The expression of different VER isoforms influences the formation of the extracellular matrix network and might modulate cell-matrix and cell-cell interactions [19]. Moreover, RNA splicing patterns suggest distinct functions for different domains of the protein. V0/V1 isoforms show similar properties with respect to cell anti-adhesion, proliferation and growth, and resistance to apoptosis. V2 is known to inhibit cell growth and proliferation, and to be expressed only in the nervous system, as the major isoform in adult brain [25]. The truncated isoform V3, sometimes called "versicant", is thought to possess pro-adhesive properties, due to the lack of the highly negatively charged chondroitin sulfate side chains [26].

Previous reports describe changes in the distribution of HA and VER in human and murine cervix during late pregnancy and parturition [27,28]. In the female rodent reproductive system, VER distribution and the expression of its isoforms were shown for the first time by Russel *et al. *[29] in the mouse ovary. However, little is known about VER expression and function in the non-pregnant uterus. The major objectives of the present study were (*i*) to analyze whether E2 and P4 modulate the expression and distribution of VER in the uterine tissues of mice and (*ii*) to characterize VER isoforms in the mouse uterus.

## Methods

### Tissue collection

Forty two Swiss female mice, aged 3–5 months, were used. Animals were housed in a 12-h light: 12-h dark, temperature-controlled (22°C) environment, with free access to food and water. The stages of the estrous cycle were determined by vaginal smears. Animals in proestrus, estrus, metaestrus and diestrus, and ovariectomized animals submitted or not to hormone replacement were anesthetized with an intraperitoneal injection of tribromoethanol (Avertin^®^) (Aldrich Chemical Company, Inc., Milwaukee, Wisconsin, USA; 0,025 mL/g body weight). The uteri were subsequently removed, cut with razor blades and immediately immersed in a fixative solution or in RNAlater solution and stored at -20°C. National guidelines for laboratory animal care were followed, and all experiments were approved by the Institute of Biomedical Sciences Animal Ethics Committee (authorization number, 144/2002).

### Ovariectomy and hormone replacement

The general protocol adopted here was adapted from Domino and Hurd [30]. Three different doses for 17β-estradiol (E2; 1, 10 and 100 μg) and medroxyprogesterone acetate (MPA; 0.25, 0.5 and 1 mg) were tested. Vaginal smear features and uterine morphology were used to establish the most appropriate protocol.

1. Tissue collection twenty days after ovariectomy.

2. Pre-treatment with daily priming doses of E2 (Sigma-Aldrich, St. Louis, Missouri, USA) (10 μg/animal), diluted in mineral oil (Schering-Plough), for three days, followed by a two-day rest and daily injections of E2 (10 μg/animal) during four consecutive days.

3. Pre-treatment with daily priming doses of E2, followed by a resting period and daily injections of MPA (500 μg/animal) (Pharmacia & Upjohn), diluted in distilled water, during four consecutive days.

4. Same pre-treatment and resting period as the previous groups, followed by daily injections of both E2 and MPA during four consecutive days.

5. Control group received injections of vehicle alone (mineral oil) for three days, followed by a two-day rest and daily oil injections during four consecutive days.

All injections were sub-cutaneous in a 100 μl volume. Twenty four hours after the last injection, the mice were anesthetized and the uteri were collected as described above.

### Light microscopy processing

Samples were fixed at 4°C for 3 h in Methacarn (absolute methanol, chloroform and glacial acetic acid; 6:3:1), rinsed with absolute ethanol, and embedded in Paraplast (Oxford, St. Louis, Missouri, USA) at 60°C. Samples were cut into 5 μm sections, adhered to glass slides using 0.1% poly-L-lysine (Sigma, St. Louis, Missouri, USA) and then dried at 37°C.

### Immunohistochemistry

Immunohistochemistry was performed according to a previously established protocol [17]. Briefly, sections were deparaffinized, hydrated and treated with 3% (v/v) H_2_O_2 _in PBS (30 min) to block endogenous peroxidase activity. They were subsequently incubated with chondroitinase ABC from *proteus vulgaris *(Seikagaku, Tokyo, Japan), diluted in 20 mM Tris-HCl buffer pH 6.0 (1 h at 37°C), prior to incubation with primary antibody. Nonspecific staining was blocked by incubating the sections (1 h) with normal goat serum, diluted 1:1 (v/v) in PBS – 10% BSA (w/v) (room temperature). Rabbit anti-VER polyclonal antibody (Chemicon International, Temecula, CA, USA) recognizing isoforms V0 and V1 was diluted 1:500 in PBS – 0.3% (v/v) Tween 20 and incubated overnight (4°C). The sections were then incubated with biotin-conjugated secondary anti-rabbit IgG (1:2000) (Rockland, Gilbertsville, Pennsylvania, USA), diluted in PBS (v/v) (1 h at room temperature) and with streptavidin-peroxidase ABC complex (Vector Labs, Burlingame, California, USA) (1 h at room temperature), according to the manufacturer's instructions. Peroxidase was visualized using 0.03% (w/v) 3,3'-diaminobenzidine in PBS with 0.03% (v/v) H_2_O_2_. The sections were counterstained with Mayer's haematoxylin. Negative controls included replacing the primary antibodies with the respective non-immune serum at similar concentrations or omitting the primary antibody step from the protocol.

A Nikon Eclipse E600 microscope was used for examining sections. Images were captured using a digital camera (Cool SNAP-Procf color; Roper Scientific, Trenton, New Jersey, USA) and Image Pro Plus software (Media Cybernetics, Silver Spring, Maryland, USA).

### mRNA extraction and semi-quantitative real-time PCR

For the molecular biology experiments, the phases of estrus – highest estrogen levels – and diestrus – highest progesterone levels – were chosen to represent the estrous cycle.

Uteri samples (n = 5) were immersed in RNAlater solution and stored at -20°C. Upon use, they were crushed in a steel mortar and pestle set (Fisher Scientific International, Inc, Hampton, New Hampshire, USA) precooled in dry ice. The crushed samples were transferred to sterile microfuge tubes and total RNA was extracted using Trizol reagent (Invitrogen, Calbard, California, USA) following the manufacturer's instructions. Reverse transcription was performed with AffinityScript QPCR cDNA Synthesis kit (Stratagene, Cedar Creek, TX, USA) and 1 μg of total RNA. The cDNA samples were used to determine which VER isoforms were expressed in the mouse uterus by PCR amplification using the Platinum Taq DNA Polymerase kit (Invitrogen) and specific primers [29]. GAPDH was used as internal control and Universal Mouse Reference RNA (Stratagene) was used as positive control in the PCR amplification experiments. The relative expression of VER mRNA was determined as described previously [31]. The relative levels of mRNA of the tested gene were estimated in duplicate samples by fluorescence quantified with the ABI Prism 7500 sequence detector (Applied Biosystems). Reactions were performed in a total volume of 25 μl containing 10 ng of cDNA and 450 nM primers in a reaction buffer containing SYBR Green PCR master mix (Stratagene). All *C*_*t *_values were normalized using GAPDH and the results were expressed as fold-induction relative to the expression of the control, the calibrator sample, arbitrarily set to 1. Primer sequences are given in Table 1.

**Table 1:** Mouse oligonucleotide primers (5'→3')

**Gene**	**Forward (F) and Reverse (R) primers**	**Amp. size**	**Genbank #**
*GAPDH	F:^848^*TCTGAGGGCCCACTGAAG*^865^; R:^1047^*AGGGTTTCTTACTCCTTGGAGG*^1068^	221 bp	NM_001001303
			
*Versican	F:^9532^*TCCTGATTGGCATTAGTGAAG *^9552^; R:^9672^*CTGGTCTCCGCTGTATCC *^9689^	158 bp	NM_001081249
			
V0	F:^3759^*TTCACAGAACGCCACCCTTGAGTCC*^3783^; R:^4363^*CTAGCTTCTGCAGCTTCCGGGTCC *^4386^	628 bp	NM_28599
			
V1	F:^1031^*GCAGCTTGGAGAAATGGCTTTGACC*^1055^; R:^4363^*CTAGCTTCTGCAGCTTCCGGGTCC *^4386^	443 bp	NM_28599
			
V2	F:^3856^*TCCTGGAGAATCTGTAACACAGCACCC*^3882^; R:^9364^*CTCGGTAGGATAACAGGTGCCTCCG*^9388^	307 bp	NM_28599
			
V3	F:^1024^*ACTTCAGGCAGCTTGGAGAAATGGC*^1048^; R:^9410^*ACTGGTCTCCGCTGTATCCAGGTGC*^9434^	302 bp	NM_28599

V0, V1, V2 and V3 are alternative splicing variants of the versican gene. *primers used for real-time PCR analysis.

### Statistical analysis

The unpaired t test was used to determine significant differences between groups and was performed using Prism 3.0 (GraphPad Software, San Diego, California, USA). Multiple comparisons were performed by ANOVA followed by the Student-Newman-Keuls test.

## Results

Two morphologically distinct compartments can be identified in the endometrial stroma, herein denoted as superficial and deep, as described previously [17].

### Immunolocalization of versican

**Figure 1:**
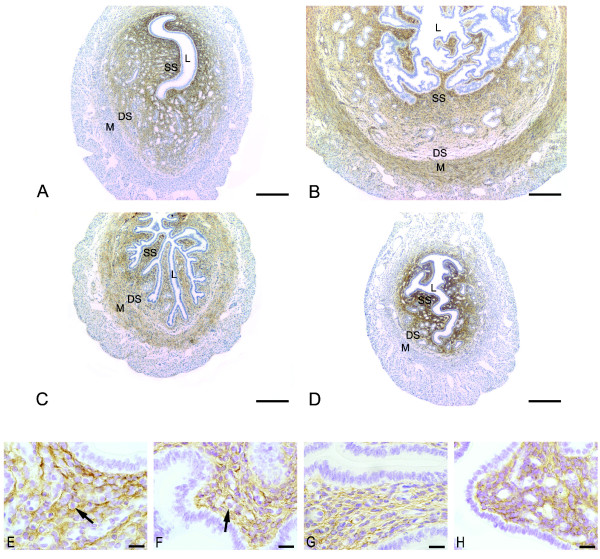
**Immunoperoxidase for versican**. (A) proestrus and (D) diestrus: the immunoreaction is seen as a dense network in the endometrial stroma, but absent in the myometrium. In both groups, the immunolabeling is stronger in the superficial stroma (SS) and only traces are observed in the interface between deep stroma (DS) and myometrium (M); (B) estrus and (C) metaestrous: the immunoreaction is observed in both superficial (SS) and deep stroma (DS). In these phases versican is also immunodetected in the internal layer of the myometrium (M); L: Lumen; SS: Superficial Stroma; DS: Deep Stroma; M: Myometrium. Scale bar: 200 μm. Higher Magnification micrographs show the localization of VER inside and outside the cells in proestrus (E), estrus (F) and diestrus (H), and mostly outside the cells in metaestrus (G). The reaction is absent from immune cells cytoplasm (arrows). Scale bar: 20 μm.

In proestrus (Figure 1A and 1E) and diestrus (Figure 1D and 1H), the immunoreaction for VER was observed as a dense network in the endometrial stroma, but was absent in the myometrium. In both groups, especially diestrus, staining was stronger in the superficial stroma with only traces at the interface between the deep stroma and the myometrium.

In estrus (Figure 1B and 1F) and metaestrus (Figure 1C and 1G), the immunoreaction was present in both superficial and deep stroma, as well as in the internal layer of the myometrium. The reaction seemed to be weaker in metaestrus.

**Figure 2:**
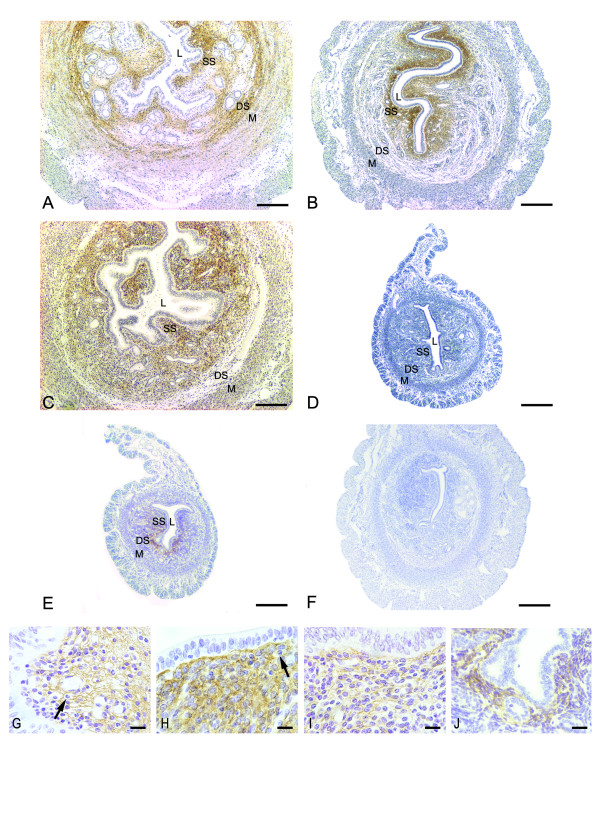
**Immunoperoxidase for versican**. (A) E2 treatment: strong immunoreaction is present in the whole endometrial stroma, except in areas of edema. The reaction can be observed as a network of delicate filaments. The immunolabeling is also observed at the interface between the deep stroma (DS) and the myometrium (M), as well as in the myometrial internal layer; (B) MPA treatment: versican distribution is seen as a dense brownish network in the extracellular spaces, exclusively in the superficial stroma (SS); (C) E2+MPA treatment: the immunoreaction is observed as a network of thin filaments in the whole endometrial stroma, except at the interface between the deep stroma (DS) and the myometrium (M). (D) ovariectomized group: immunoreaction is absent from the uterine tissues; (E) oil control group: immunoreaction is seen underneath the luminal epithelium of the antimesometrial stroma. The negative control shows no immunoreaction (F). L: Lumen; SS: Superficial Stroma; DS: Deep Stroma; M: Myometrium. Scale bar: 200 μm. Higher Magnification micrographs show the localization of VER inside and outside the cells in the MPA (H) and oil (J) groups, and mostly outside the cells in the E2 (G) and E2+MPA (I) groups. The reaction is absent from immune cells cytoplasm (arrows). Scale bar: 20 μm.

In the E2-treated group, strong immunoreaction was present in the whole endometrial stroma, except in areas of edema. The reaction was observed as a network in the intercellular spaces. Staining was also observed at the interface between the deep stroma and the myometrium, as well as in the myometrial internal layer (Figure 2A and 2G).

In the MPA-treated group, VER was seen exclusively in the superficial stroma as a dense network in the extracellular spaces, as well as in the cytoplasm of endometrial fibroblasts (Figure 2B and 2H).

In the E2+MPA-treated group, VER was present as a network of thin filaments in the ECM in the whole endometrial stroma, except at the interface between the deep stroma and the myometrium of the antimesometrial region. The immunoreaction was present in both layers of the myometrium (Figure 2C and 2I).

In ovariectomized (ovx) animals (Figure 2D), VER immunoreactivity was absent from uterine tissues. In the oil-control group (Figure 2E and 2J), immunoreaction was present in a narrow area of the superficial stroma, mainly in the antimesometrial region.

### Relative expression of versican mRNA

**Figure 3:**
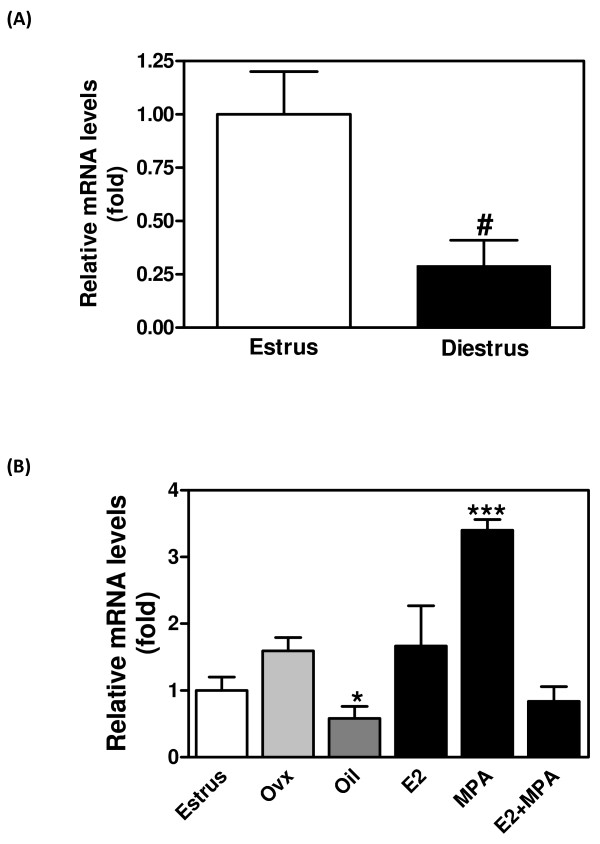
**Relative mRNA expression of versican in the mouse uterus**. The mRNA expression was analyzed by real-time PCR using primers common to all splice variants, and relative gene expression determined by designating estrus to 1. (A) versican mRNA in estrus and diestrus; (B) versican mRNA after ovariectomy and hormone replacement. Values represent the mean + SE of determinations on three independent tissue preparations. # *p *< 0.05 vs. estrus by Student t-test; *p < 0.05 vs. ovx and E2; ** p < 0.001 vs. all by ANOVA.

VER mRNA was estimated by real time PCR of uterine cDNA obtained during the estrous cycle or after ovariectomy or hormone replacement (n = 5). Figure 3A shows that the relative expression of VER was ~3 fold higher in estrus than in diestrus (p < 0.05). After ovariectomy, VER mRNA was increased in the MPA-treated group (~2 fold; p < 0.001). The treatment with E2 or E2+MPA did not significantly alter VER mRNA, if compared to estrus. However, oil injections significantly decreased VER mRNA expression when compared to Ovx and E2 (Figure 3B).

### Expression of versican isoforms

**Figure 4:**
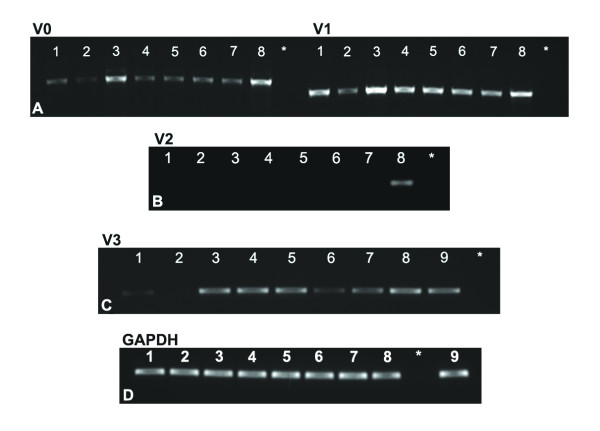
**Expression of versican isoforms**. Gel analysis of products generated by RT-PCR using specific primer sets for versican V0, V1, V2 and V3. Note the presence of V0 (left panel), V1 (right panel) (A) and V3 (C), but the absence of V2 (B). GAPDH internal control shows uniform expression in all groups studied (D). Total RNA isolated from whole mouse uterus from all groups studied (n = 5). 1- Diestrus; 2- Estrus; 3- E2; 4- MPA; 5- E2+MPA; 6- ovx; 7- oil; 8- UMRR (Universal Mouse Reference RNA); 9- ovary; * – negative control.

mRNA analysis (Figure 4A–C) showed that V0, V1 and V3 are all expressed in the uterus. V2 was not detected in the estrous cycle or following hormone treatment. In general, V1 showed the most uniform expression across treatments. V3 was not detected in estrus. GAPDH was used as internal control (Figure 4D).

## Discussion

Whereas E2 levels are high throughout the estrous cycle, and highest in estrus, P4 levels are very low in estrus and peak in diestrus [6]. Several reports have correlated these fluctuations with alterations in the expression of components of the uterine ECM, including glycosaminoglycans, proteoglycans and growth factors [32-34]. The present data show that the distribution and expression of VER in the mouse uterus are highly sensitive to estrous cycle stage, with intricate modulation of deposition in different tissue layers. Furthermore, hormone replacement studies have revealed that exogenous estrogen and combined estrogen/progestin have profound influences on the ECM that differ in different tissue layers.

Immunolocalization shows VER in endometrial ECM in both proestrus and estrus, with undetectable levels in myometrium in proestrus and clearly increasing levels in myometrium in estrus. This may indicate a basal level of transcription in unstimulated endometrial stromal cells, in keeping with the presence of mRNA in uterine tissue from ovariectomized animals. Ovariectomy leads to loss of VER deposition in both endometrium and myometrium. The oil control group shows a discrete immunoreaction restricted to a narrow area of the superficial stroma mainly in the antimesometrial stroma. This discrete protein deposition may be related to basal levels of estrogen produced by the adrenal glands that remain after ovariectomy. In addition, there is a constitutive expression of ER and PR even after ovariectomy [35]. Increasing estrogen appears to stimulate the deposition of VER in the ECM of the inner myometrial layer at estrus. In keeping with this idea, after estrogen replacement, VER is also seen in the endometrial stroma and inner layer of the myometrium. Thus estrogen has a powerful effect on VER in uterine ECM. Surprisingly, however, VER mRNA is maintained at equal levels in ovariectomized control uterus and after estrogen stimulation. It is possible that changes in VER mRNA in different layers may be obscured in the whole-tissue mRNA analysis. However, it is equally possible that estrogenic control of VER is not exerted through mRNA abundance. Versican expression may be regulated by specific proteases, as previously demonstrated in other models [36]. ADAMTS-1 and 4 are versicanases, and are regulated by sexual hormones [37]. Moreover, ADAMTS-1, 4 and 5 are modulated in the mouse ovary and ADAMTS-1 is present in the mouse uterus [38,39]. Also ECM changes occur on a longer timescale than those generally seen in intracellular compartments.

In diestrus there is a striking change in the myometrium which is now VER-negative, while the protein remains detectable in endometrium. At mRNA level there is a marked drop in abundance, suggesting that before diestrus, myometrial mRNA was quantitatively dominant. These data suggest that P4 in combination with estradiol acts to inhibit VER transcription in myometrium, adding supporting evidence that hormone responses in this tissue compartment are distinct from those in endometrium. After the hormone replacement regime in which estradiol was followed either by MPA, or MPA in combination with estradiol, quite different effects were seen. MPA alone produced strong VER expression in the superficial endometrial stroma, no staining in the deep stroma or myometrium, and yet a doubling of total mRNA relative to the level seen in control animals. Since estrogen alone produces relatively uniform VER deposition in both endometrium and myometrium, this series of experiments may suggest that MPA stimulates loss of VER from ECM, and that this may be followed by *de novo *synthesis in the superficial stroma, with high levels of VER mRNA in this layer alone. In this situation it appears that endometrial mRNA must be quantitatively dominant. Additionally, in diestrus, proestrus and MPA groups, the immunoreaction is observed in the ECM, as well as inside the cells, whereas in metaestrus, E2 and E2+MPA groups most of the reaction is seen outside the cells of the endometrial stroma, suggesting a kinetics of synthesis and secretion orchestrated by ovarian hormones.

The presence of V0, V1 and V3 mRNA variants was also shown in the mouse uterus. mRNA encoding the V3 isoform becomes undetectable in the estrus phase, supporting a general conclusion that VER mRNA is altering dynamically during the cycle, and providing evidence for hormonal regulation of mRNA splicing. Other workers have detected VER isoforms in reproductive tract tissues: Dours-Zimmermann and Zimmermann [40] showed V0 and V1 in human myometrium. VER expression is very low in the cervix of non-pregnant women, but increases considerably in pre-term pregnancy and at labor [27]. Recently, Ruscheinsky *et al*. [28] studied the expression of HA and VER in the mouse cervix and evidenced V1 as the most abundant isoform. There is no clear evidence that V0 and V1 play distinct roles and they are generally co-expressed [41]. Isoforms with the exception of V2 were found in the mouse and bovine ovary [29,42]. The V3 isoform lacks the CS attachment sites and its role in the ECM is likely to be distinct from V0 and V1. However, available antibodies do not recognize V3, so the staining reported here must be regarded as representing only V0 and V1. Further work is required to define the distribution of the V3 protein isoform and its functional role.

Uterine stroma is compartmentalized into superficial and deep zones with distinct cell populations [17,43]. Indeed, morphological differences observed between rounded cells in the superficial stroma and spindle-shaped cells in the deep stroma may be related to the formation of a VER-rich pericellular matrix in the former environment. ECM may influence the tension exerted on cells, in turn affecting cell shape and behavior [20,44]. *In vitro *experiments have revealed that HA- and VER-rich pericellular matrix is related to cell detachment and mitotic cell rounding [45]. Versican accumulation in the superficial stroma in diestrus and after MPA treatment matches high levels of proliferating cells (data not shown).

Furthermore, the transdifferentiation of endometrial fibroblasts into decidual cells requires the expression of connexins and gap junction formation [46]. Recent studies showed that VER is able to enhance the expression of connexin 43 *in vitro *[47]. As versican is expressed at the maternal-fetal interface, we may speculate a role for this proteoglycan in embryo implantation.

Importantly, MPA activates both the progesterone and androgen receptors. Androgen receptor is expressed in mouse uterine stroma [48] and has been shown to play a role in decidualization in synergy with the PR [49]. Since androgens are likely to be active during the estrous cycle along with progesterone, MPA is an appropriate choice of ligand for hormone replacement studies. In addition, a previous study demonstrated that proper decidualization occurs when using MPA to stimulate decidual differentiation [50]. However, its effects are not interpretable solely in terms of actions at the PR.

Another variable in the system that requires further attention is VER mRNA half-life, as steroid hormones are able to regulate gene expression by altering the stability of mRNAs and the ratio of activity of RNAses and their inhibitors [51-53]. Furthermore, hormone regulation of splicing, the kinetics of translation and post-translational modification, kinetics of ECM deposition and localization, and the regulation and activation of proteases and glycosidases that degrade VER must all be considered. However, we anticipate that the dramatic hormonal effects unveiled in the present study will stimulate further investigations of these regulatory events in the ECM and their role in preparing the uterus to accept and support a developing embryo.

## Conclusion

In conclusion, the distribution and expression of VER in the mouse uterus are highly sensitive to estrous cycle stage and modulated by estrogen and progesterone, each hormone acting in a tissue specific manner. Furthermore, hormonal control of VER appears not to be exerted only through mRNA abundance. Other factors such as specific proteases, as well as mRNA stability, may be modulated by ovarian hormones.

## Competing interests

We hereby declare that there is no conflict of interest that could be perceived as prejudicing the impartiality of the research reported in this manuscript.

## Authors' contributions

RS carried out the collection and preparation of samples, the staining procedures, part of the molecular biology experiments and drafted the manuscript. LC carried out most of the molecular biology experiments and participated in the analysis and interpretation of real time PCR data. RF contributed in revising the manuscript critically, besides participating in the animals' treatment experiments. JG participated in the molecular biology experiments, contributed in the analysis and interpretation of data and helped to draft the manuscript. JA has made substantial contributions to analysis and interpretation of data and helped to draft the manuscript, adding important intellectual content. TZ conceived of the study, participated in its design and coordination and helped to draft the manuscript. All authors read and approved the final manuscript.

## References

[B1] Allen E (1927). The oestrous cycle in the mouse. Am J Anat.

[B2] Martin L, Finn CA (1969). Hormone secretion during early pregnancy in the mouse. J Endocrinol.

[B3] Parandoosh Z, Crombie DL, Tetzke TA, Hayes JS, Heap RB, Wang M-W (1995). Progesterone and oestrogen receptors in the decidualized mouse uterus and effects of different types of anti-progesterone treatment. J Reprod Fertil.

[B4] Lydon JP, DeMayo FJ, Funk CR, Mani SK, Hughes AR, Montgomery CA, Shyamala G, Conneely OM, O'Malley BW (1995). Mice lacking progesterone receptor exhibit pleiotropic reproductive abnormalities. Genes & Development.

[B5] Weihua Z, Saji S, Sirpa M, Cheng G, Jensen EV, Warner M, Gustafsson J (2000). Estrogen receptor (ER) β, a modulator of ERα in the uterus. Procl Natl Acad Sci USA.

[B6] Wood GA, Fata JE, Watson KLM, Khokha R (2007). Circulating hormones and estrous stage predict cellular and stromal remodeling in murine uterus. Reproduction.

[B7] Kresse H, Schönherr E (2001). Proteoglycans of the extracellular matrix and growth control. J Cell Physiol.

[B8] Aplin JD, Charlton AK, Ayad S (1988). An immunohistochemical study of human endometrial extracellular matrix during the menstrual cycle and first trimester of pregnancy. Cell Tissue Res.

[B9] Iwahashi M, Muragaki Y, Ooshima A, Yamoto M, Nakano R (1996). Alterations in distribution and composition of the extracellular matrix during decidualization of the human endometrium. J Reprod Fertil.

[B10] Aplin JD, Glasser SR, Aplin JD, Giudice LC, Tabibzadeh S (2002). Endometrial Extracellular Matrix. The Endometrium.

[B11] Zorn TM, Bevilacqua EM, Abrahamsohn PA (1986). Collagen remodeling during decidualization in the mouse. Cell Tissue Res.

[B12] Teodoro WR, Witzel SS, Velosa AP, Shimokomaki M, Abrahamsohn PA, Zorn TM (2003). Increase of interstitial collagen in the mouse endometrium during decidualization. Connect Tissue Res.

[B13] Carbone K, Pinto NM, Abrahamsohn PA, Zorn TM (2007). Arrangement and fine structure of collagen fibrils in the decidualized mouse endometrium. Microsc Res Tech.

[B14] Spiess K, Zorn TM (2007). Collagen types I, III, and V constitute the thick collagen fibrils of the mouse decidua. Microsc Res Tech.

[B15] Greca CP, Abrahamsohn PA, Zorn TMT (1998). Ultrastructural cytochemical study of proteoglycans in the endometrium of pregnant mice using cationic dyes. Tissue Cell.

[B16] San Martin S, Soto-Suazo M, Ferreira de Oliveira S, Aplin JD, Abrahamsohn P, Zorn TMT (2003). Small leucine-rich proteoglycans (SLRPs) in uterine tissues during pregnancy in mice. Reproduction.

[B17] Salgado RM, Favaro RR, San Martin S, Zorn TM (2009). The estrous cycle modulates small leucine-rich proteoglycans expression in mouse uterine tissues. Anat Rec (Hoboken).

[B18] San Martin S, Soto-Suazo M, Zorn TMT (2003). Distribution of versican and hyaluronan in the mouse uterus during decidualization. Braz J Med Biol Res.

[B19] Shinomura T, Zako M, Ito K, Ujita M, Kimata K (1995). The gene structure and organization of mouse PG-M, a large chondroitin sulfate proteoglycan. J Biol Chem.

[B20] Wight TN (2002). Versican: a versatile extracellular matrix proteoglycan in cell biology. Curr Opin Cell Biol.

[B21] Zimmermann D, Iozzo R (2000). Versican. Proteoglycans – Structure, Biology and Molecular Interactions.

[B22] Isogai Z, Aspberg A, Keene DR, Ono RN, Reinhardt DP, Sakai LY (2002). Versican interacts with fibrillin-1 and links extracellular microfibrils to other connective tissue networks. J Biol Chem.

[B23] Stumm CL, Zorn TM (2007). Changes in fibrillin-1 in the endometrium during the early stages of pregnancy in mice. Cells Tissues Organs.

[B24] Wu YJ, La Pierre DP, Wu J, Yee AJ, Yang BB (2005). The interaction of versican with its binding partners. Cell Res.

[B25] Schmalfeldt M, Dours-Zimmermann MT, Winterhalter KH, Zimmermann DR (1998). Versican V2 is a major extracellular matrix component of the mature bovine brain. J Biol Chem.

[B26] Zako M, Shinomura T, Ujita M, Ito K, Kimata K (1995). Expression of PG-M(V3), an alternatively spliced form of PG-M without a chondroitin sulfate attachment region in mouse and human tissues. J Biol Chem.

[B27] Westergren-Thorsson G, Norman M, Bjornsson S, Endresen U, Stjernholm Y, Ekman G, Malmstrom A (1998). Differential expressions of mRNA for proteoglycans, collagens and transforming growth factor-beta in the human cervix during pregnancy and involution. Biochem Biophys Acta.

[B28] Ruscheinsky M, De la Motte C, Mahendroo M (2008). Hyaluronan and its binding proteins during cervical ripening and parturition: dynamic changes in size, distribution and temporal sequence. Matrix Biol.

[B29] Russell DL, Ochsner SC, Hsieh M, Mulders S, Richards JS (2003). Hormone-regulated expression and localization of versican in the rodent ovary. Endocrinology.

[B30] Domino SE, Hurd EA (2004). LacZ expression in Fut2-LacZ reporter mice reveals estrogen-regulated endocervical glandular expression during estrous cycle, hormone replacement, and pregnancy. Glycobiology.

[B31] Capelo LP, Beber EH, Huang SA, Zorn TM, Bianco AC, Gouveia CH (2008). Deiodinase-mediated thyroid hormone inactivation minimizes thyroid hormone signaling in the early development of fetal skeleton. Bone.

[B32] Takata K, Teramaya H (1977). Hormonal effect on glycosaminoglycans and glycoproteins in uteri of ovariectomized rats. Biochim Biophys Acta.

[B33] Wu WX, Zhang Q, Unno N, Derks JB, Nathanielsz PW (2000). Characterization of decorin RNAm in pregnant intrauterine tissues of the ewe and regulation by steroids. Am J Physiol Cell Physiol.

[B34] Gaide Chevronnay HP, Cornet PB, Delvaux D, Lemoine P, Courtoy PJ, Henriet P, Marbaix E (2008). Opposite regulation of transforming growth factors-beta2 and -beta3 expression in the human endometrium. Endocrinology.

[B35] Tibetts TA, Mendoza-Meneses M, O'Malley BW, Conneely OM (1998). Mutual and intercompartmental regulation of estrogen receptor and progesterone receptor expression in the mouse uterus. Biol Reprod.

[B36] Sandy JD, Westling J, Kenagy RD, Iruela-Arispe ML, Verscharen C, Rodriguez-Mazaneque JC, Zimmermann DR, Lemire JM, Fischer JW, Wight TN, Clowes AW (2001). Versican V1 proteolysis in human aorta in vivo occurs at the Glu441-Ala442 bond, a site that is cleaved by recombinant ADAMTS-1 and ADAMTS-4. J Biol Chem.

[B37] Russell DL, Doyle KM, Ochsner SA, Sandy JD, Richards JS (2003). Processing and localization of ADAMTS-1 and proteolytic cleavage of versican during cumulus matrix expansion and ovulation. J Biol Chem.

[B38] Kim J, Kim H, Lee SJ, Choi YM, Lee SJ, Lee JY (2005). Abundance of ADAM-8, -9, -10, -12, -15 and -17 and ADAMTS-1 in mouse uterus during the oestrous cycle. Reprod Fertil Dev.

[B39] Richards JS, Hernandez-Gonzalez I, Gonzalez-Robayna I, Teuling E, Lo Y, Boerboom D, Falender AE, Doyle KH, LeBaron RG, Thompson V, Sandy JD (2005). Regulated expression of ADAMTS family members in follicles and cumulus oocyte complexes: evidence for specific and redundant patterns during ovulation. Biol Reprod.

[B40] Dours-Zimmermann MT, Zimmermann DR (1994). A novel glycosaminoglycans attachment domain identified in two alternative splice variants of human versican. J Biol Chem.

[B41] LeBaron RG (1996). Versican. Perspect Dev Neurobiol.

[B42] Irving-Rodgers HF, Catanzariti KD, Aspden WJ, D'Occhio MJ, Rodgers RJ (2006). Remodeling of extracellular matrix at ovulation of the bovine ovarian follicle. Mol Reprod Dev.

[B43] Oliveira SF, Abrahamsohn P, Zorn TM (1998). Autoradiography reveals regional metabolic differences in the endometrium of pregnant and nonpregnant mice. Braz J Med Biol Res.

[B44] Chicurel ME, Chen CS, Ingber DE (1998). Cellular control lies in the balance of forces. Curr Opin Cell Biol.

[B45] Evanko SP, Angello JC, Wight TN (1999). Formation of hyaluronan and versican-rich pericellular matrix is required for proliferation and migration of vascular smooth muscle cells. Arterioscler Thromb Vasc Biol.

[B46] Abrahamsohn PA, Zorn TMT (1993). Implantation and decidualization in rodents. Journal of Experimental Zoology.

[B47] Sheng W, Dong H, Lee DY, Lu WY, Yang BB (2007). Versican modulates gap junction intercellular communication. J Cell Physiol.

[B48] Pelletier G, Luu-The V, Li S, Labrie F (2004). Localization and estrogenic regulation of androgen receptor mRNA expression in the mouse uterus and vagina. J Endocrinol.

[B49] Cloke B, Huhtinen K, Fusi L, Kajihara T, Yliheikkilä M, Ho KK, Teklenburg G, Lavery S, Jones MC, Trew G, Kim JJ, Lam EW, Cartwright JE, Poutanen M, Brosens JJ (2008). The androgen and progesterone receptors regulate distinct gene networks and cellular functions in decidualizing endometrium. Endocrinology.

[B50] Akcali KC, Khan SA, Moulton BC (1996). Effect of Decidualization on the Expression of bax and bcl-2 in the Rat Uterine Endometrium. Endocrinology.

[B51] Ing NH (2005). Steroid hormones regulate gene expression posttranscriptionally by altering the stabilities of messenger RNAs. Biol Reproduction.

[B52] Schauer RC (1991). Effects of estradiol and progesterone on rat uterine ribonuclease inhibitor activity. Horm Metab Res.

[B53] Rao KS, Sirdeshmukh R, Gupta PD (1994). Modulation of cytosolic RNase activity by endogenous RNase inhibitor in rat vaginal epithelial cell on estradiol administration. FEBS Lett.

